# Induction of whole-body gene deletion *via* R26-regulated tamoxifen-inducible Cre recombinase activity

**DOI:** 10.3389/fphar.2022.1018798

**Published:** 2022-12-08

**Authors:** Rahul Kumar, Yun Mao, Sonika Patial, Yogesh Saini

**Affiliations:** Department of Comparative Biomedical Sciences, School of Veterinary Medicine, Louisiana State University, Baton Rouge, LA, United States

**Keywords:** tamoxifen, Cre-ERT2, inducible gene deletion, Cre recombinase, reporter mice

## Abstract

Germline deletion of certain genes causes embryonic lethality, therefore, understanding the effect of deletion of such genes on mammalian pathophysiology remains challenging. Tamoxifen (TAM)-inducible Cre recombinase is widely used for tissue-specific and temporal induction of gene deletion in mice. However, the tamoxifen treatment regimen for the generation of whole-body deletion of a gene is not yet fully standardized for the majority of organs/tissues. Accordingly, we employed GtROSA26 (R26) promoter-regulated Cre and a reporter gene expression strategy. GtROSA26 (R26) is an ubiquitous promoter and mice carrying the R26^Cre−ERT2^ transgene express Cre-ERT2 in all the cells. Similarly, mice carrying the R26^mTOM-mEGFP^ transgene express mTOM (membrane-targeted tdTomato), in the absence of Cre or mEGFP (membrane-targeted enhanced green fluorescent protein), in the presence of Cre, in all the cells. The progeny carrying one allele of both transgenes were subjected to different TAM regimens, i.e., IP injections (4 injections; 1.35 mg/injection), diet (400 mg TAM-citrate/kg food), or diet (400 mg TAM-citrate/kg food) combined with either TAM-oral gavage (4 gavages; 1.35 mg/gavage) or TAM IP injections (4 injections; 1.35 mg/injection) for 2-weeks beginning at postnatal day (PND) 21 and the extent of Cre recombination in different tissues was determined at PND35. Tamoxifen administration resulted in a transient loss of body weight in all the treatment regimens with a relatively slower rate of weight gain in the TAM-diet plus TAM-oral gavage group compared to other groups. While the efficiency of Cre recombination, as determined by the expression of mEGFP protein, was variable among tissues, major tissues such as the liver, heart, lungs, spleen, and thymus—showed almost complete recombination. No recombination was evident in any of the tissues examined from the control mice. In general, the efficiency of Cre recombination was better with a combined regimen of TAM-diet with either TAM-injections or TAM-oral gavage compared to TAM-diet alone or TAM-injections alone. Our results demonstrate that a combination of TAM-diet with either TAM-injections or TAM-oral gavage can be employed for the efficient deletion of a gene in the whole body. Our findings will provide technical expertise to the researchers employing TAM-inducible Cre for the deletion of floxed genes in varied tissues.

## 1 Introduction

Germline deletion of certain genes interferes with normal fetal or perinatal development that compromises the viability of fetuses or neonates, thus making it challenging to explore the roles of such genes in mammalian pathophysiology. To circumvent these challenges, the postnatal deletion of a gene can be achieved using the inducible Cre-Lox approach (Feil et al., 2009). Tamoxifen (TAM)-inducible Cre recombinase is widely used for tissue-specific and temporal induction of gene deletion in mice. The gene deletion is typically achieved by intraperitoneal (IP) or oral administration of tamoxifen. However, the tamoxifen treatment regimen for the generation of whole-body deletion of a gene is not yet fully standardized. Furthermore, the effectiveness of TAM administration *via* these routes remains poorly characterized. Therefore, a comprehensive evaluation of the effectiveness of various TAM treatments for major body tissues is warranted.

GtROSA26 (R26) is a ubiquitously expressed locus found on mouse chromosome 6 (Friedrich and Soriano, 1991; Zambrowicz et al., 1997). It encodes a nonessential RNA in all the cells of the body. Hence, this locus can be used for the insertion of Cre recombinase or reporter transgene without causing any undesirable effects. For example, the insertion of floxed neomycin/PolyA stop cassette upstream to a toxin gene in this locus can be used to ablate certain cell lineages when recombination is induced by a cell-specific Cre recombinase (Saini et al., 2016; Saini et al., 2018). Similarly, the insertion of floxed reporter transgene to this locus allows lineage tracing when recombination is induced by cell-specific Cre recombinase (Mao et al., 2001; Srinivas et al., 2001). Cre recombinase induction can be regulated if the Cre recombinase transgene is modified to express a ligand-responsive fusion protein, i.e., Cre recombinase estrogen receptor-T2 (Cre-ERT2). The advantage of this strain is that the ERT2 moiety of the translated fusion protein restricts the Cre recombinase to the cytoplasm when ER ligand is not present. This inhibition is released upon binding of ERT2 with 4-hydroxytamoxifen, a ligand for ERT2 which, in turn, allows the translocation of Cre recombinase into the nucleus and results in recombination within the genomic LoxP sites (Feil et al., 1996; Indra et al., 1999). Thus, ligand-dependent Cre recombinase activity offers temporal control over gene deletion.

The R26 locus can also be modified by the insertion of Cre recombinase transgene which can be used to induce global recombination in floxed genomic alleles (Vooijs et al., 2001; Seibler et al., 2003). Mice carrying Cre-ERT2 transgene that is targeted to the R26 locus have been generated (Ventura et al., 2007). The R26^mTOM-mEGFP^ reporter mouse contains R26-regulated dual-fluorescence (mTOM-mEGFP; mTmG) transgene which can be used as a readout of Cre recombinase activity (Muzumdar et al., 2007). The mTmG construct contains a proximal LoxP-flanked membrane-targeted tdTomato (mTOM)/polyA stop cassette and a distal membrane-targeted enhanced green fluorescent protein (mEGFP) transgene. This gene construct allows exclusive expression of membrane-localized mTOM protein and prevents the expression of membrane-localized mEGFP when Cre recombinase is absent. The expression of Cre recombinase, however, mediates the excision of the floxed region that prevents mTOM expression but enables mEGFP expression. Thus, the latter acts as a readout of Cre recombinase activity.

We conceived this study to establish and standardize a postnatal inducible global gene deletion approach. To achieve this, R26^Cre−ERT2/Cre−ERT2^ mice, which express R26-regulated Cre-ERT2, were crossed with R26^mTOM-mEGFP/mTOM-mEGFP^ mice, which express R26-regulated mTOM/mEGFP, to obtain R26^Cre-ERT2/mTOM-mEGFP^ progeny expressing Cre-ERT2 as well as mTOM in all cells. The progenies were subjected to different TAM regimens and tested to determine the efficiency of inducible Cre recombination in different cells and tissues. We used three complementary approaches, i.e., fluorescence microscopy, immunohistochemical staining, and gene expression analyses. Our analyses revealed critical information regarding tissue-specific TAM-mediated recombination efficiency within a floxed transgene, which will be useful for researchers employing TAM-inducible Cre for the deletion of floxed genes in different tissues.

## 2 Materials and methods

### 2.1 Generation of inducible reporter mice

Mice expressing Cre-ERT2 under R26 promoter (R26^Cre-ERT2/Cre-ERT2^; Stock no. 008463, Jackson Laboratory, Bar Harbor, ME) (Ventura et al., 2007) were crossed with mice containing R26 promoter-regulated mTOM/mEGFP transgene (R26^mTOM-mEGFP/mTOM-mEGFP^; Stock no. 007676, Jackson Laboratory, Bar Harbor, ME) (Muzumdar et al., 2007) to generate F1 progeny expressing single copies of Cre-ERT2 and mTOM/mEGFP transgene (R26^Cre-ERT2/mTOM-mEGFP^) in all cells and tissues. The genotypes were ascertained by PCR. For R26^mTOM-mEGFP/mTOM-mEGFP^ strain, three primers (WT forward 5’-CTC TGC TGC CTC CTG GCT TCT-3’; WT Reverse 5’-CGA GGC GGA TCA CAA GCA ATA-3’; Mutant Reverse 5’-TCA ATG GGC GGG GGT CGT T-3’) were used for wild-type (WT) and mutant alleles. For R26^Cre−ERT2^ strain, four primers (WT forward 5’- CTG GCT TCT GAG GAC CG-3’; WT reverse 5’- CCG AAA ATC TGT GGG AAG TC-3’, mutant forward 5’- CGT GAT CTG CAA CTC CAG TC-3’, mutant reverse 5’- AGG CAA ATT TTG GTG TAC GG-3’) were used for wild-type (WT) and mutant alleles. PCR conditions were 93°C for 4 min followed by 38 cycles of 94°C for 20 s, 60°C for 30 s, and 72°C for 45 s, with a final extension of 72°C for 5 min. The F1 weanlings were administered with tamoxifen to drive Cre recombination and induce the systemic expression of mEGFP. All animal experiments were approved by the Louisiana State University Animal Care and Use Committee and were performed as per the standards and procedures outlined in the National Institute of Health Guide for the Care and Use of Laboratory Animals.

### 2.2 Induction of Cre recombination

The F1 weanlings (*N* = 8–10/group) were subjected to different tamoxifen (TAM) regimens, i.e., TAM intraperitoneal (IP) injections (4 IP injections @ 1.35 mg/IP injection), TAM-diet [400 mg TAM-citrate/kg food every day starting at postnatal day 21 (PND21) until postnatal day 35 (PND35)], or TAM-diet (400 mg TAM-citrate/kg food *ad libitum* starting at PND21 until PND35) combined with either TAM-oral gavage (4 oral gavages @ 1.35 mg/gavage) or TAM IP injections (4 IP injections @ 1.35 mg/IP injection). Tamoxifen (T5648) was purchased from Sigma Aldrich (Burlington, MA) and was dissolved in corn oil for IP injections or oral gavage. Tamoxifen diet (TD. 130860) was purchased from Envigo (Indianapolis, IN). Intraperitoneal injections or oral gavages were performed on four alternate days starting at PND21 until PND27. The mice from different groups were housed in separate cages and supplied with food and water *ad libitum*. The body weights of mice were recorded on alternate days until the day of necropsy and compared with cohorts maintained on a standard chow diet.

### 2.3 Fluorescence microscopy

Mice were humanely euthanized on PND35. A midline laparotomy was performed and organs including the liver, lungs, heart, spleen, thymus, kidney, and small intestine were collected. Dorsal skin was collected at the thoraco-lumbar region and the brain was collected after opening the cranial cavity. A section of all the tissues was immediately embedded in an optimal cutting temperature (OCT) medium (Sakura Finetek, Torrance, CA) and a second section was fixed in 10% neutral buffered formalin. Eight-micron thick frozen tissue sections were obtained using a cryostat, fixed in cold acetone for 20 min, washed three times with PBS, and mounted with ProLong Diamond Antifade Mountant with DAPI (P36962, Thermo Fischer Scientific). The slides were imaged in the Echo Revolve fluorescence microscope using DAPI and FITC filters to access nuclear DNA and mEGFP fluorescence, respectively (Echo, San Diego, CA).

### 2.4 Immunohistochemistry

Immunohistochemical staining for green fluorescent protein and red fluorescent protein was performed on formalin-fixed, paraffin-embedded liver, lungs, heart, spleen, thymus, kidney, brain, small intestine, and skin sections using a standard protocol as published previously (Choudhary et al., 2021a; Choudhary et al., 2021b). Briefly, tissue sections were first deparaffinized with Citrisolv (Decon Labs Inc. King of Prussia, PA) (2 × 5 min each) and then rehydrated through descending grades of ethanol (100%, 95%, 70%, 30%, distilled water; 3 min each). Antigen retrieval was performed using a citrate-based heat-induced method [heating slides in 10 mM sodium citrate (pH 6.0), containing 0.05% Tween 20, at 95°C–100°C for 30 min]. Endogenous peroxides were quenched with 3% hydrogen peroxide solution for 10 min at room temperature. Sections were blocked with blocking serum for 20 min followed by incubation in the primary antibodies [goat anti-GFP polyclonal antibody (ab111258; Abcam, Waltham, MA) and rabbit anti-RFP polyclonal antibody (600401379; Rockland Immunochemicals, Philadelphia, PA)] at room temperature for 1 h. Sections were washed and incubated with respective biotinylated secondary antibodies for 1 h at room temperature. The sections were then rinsed in deionized water (2 × 5 min each) and processed using VECTASTAIN Elite ABC HRP Kits (PK-6101/6105; Vector Laboratories, Burlingame, CA), followed by chromogenic substrate conversion using ImmPACT NovaRED HRP Substrate Kit (SK-4805; Vector Laboratories). Sections were counterstained with Gill’s Hematoxylin-I (EMD Millipore Corporation, Burlington, MA) for 5 min, rinsed in deionized water, dehydrated with ascending grades of ethanol, and cover-slipped with VectaMount mounting media (H-5000; Vector Laboratories). Immunostained slides were analyzed for positive staining and photographs were captured under the ×40 objective of the ECLIPSE Ci-L microscope with DS-Fi2 camera attachment (Nikon, Melville, NY).

### 2.5 Quantitative real-time RT-PCR

RT-qPCR for the quantification of *mTOM* and *mEGFP* was performed using the absolute quantification method (Lewis et al., 2017; Choudhary et al., 2021b). Briefly, total RNA was isolated from tissues using Illustra RNAspin Mini-RNA isolation kit, according to the manufacturer’s recommendations (GE Healthcare and Biosciences, Pittsburgh, PA). A total RNA of 0.5 µg was used for cDNA synthesis using an iScript cDNA synthesis kit (1708841; Bio-Rad, Hercules, CA). Quantitative real-time RT-PCR was performed on the ABI Prism 7900 Sequence Detection System (Applied Biosystems, Waltham, MA). Primer sequences were as follows: *mTOM*, forward 5’-GGA CAC GCT GGA CAT CA-3’, reverse 5’-CAT GCC GTA CAG GAA CAG GT-3; *mEGFP*, forward 5’-GCT ACC CCG ACC ACA TGA-3’, reverse 5’-TCT TGT AGT TGC CGT CGT CC-3’; *Actb*, forward 5’-GGC TGT ATT CCC CTC CAT CG-3’, reverse 5’-GGG GTA CTT CAG GGT CAG GA-3’. The CT values were extrapolated to copy numbers using respective standards of genes. The copy numbers of *mTOM* and *mEGFP* were normalized with that of *Actb* internal controls.

### 2.6 Statistical analysis

Statistical significance among groups was determined using ANOVA followed by Tukey’s post-hoc test for multiple comparisons. The data were expressed as means ± SEM and *p* < 0.05 was considered statistically significant. Statistical analysis was performed using GraphPad Prism version 9.4.0 (GraphPad Software, La Jolla, CA).

## 3 Results

### 3.1 Generation of R26^Cre−ERT2/mTOM/mEGFP^ reporter mice

**FIGURE 1:**
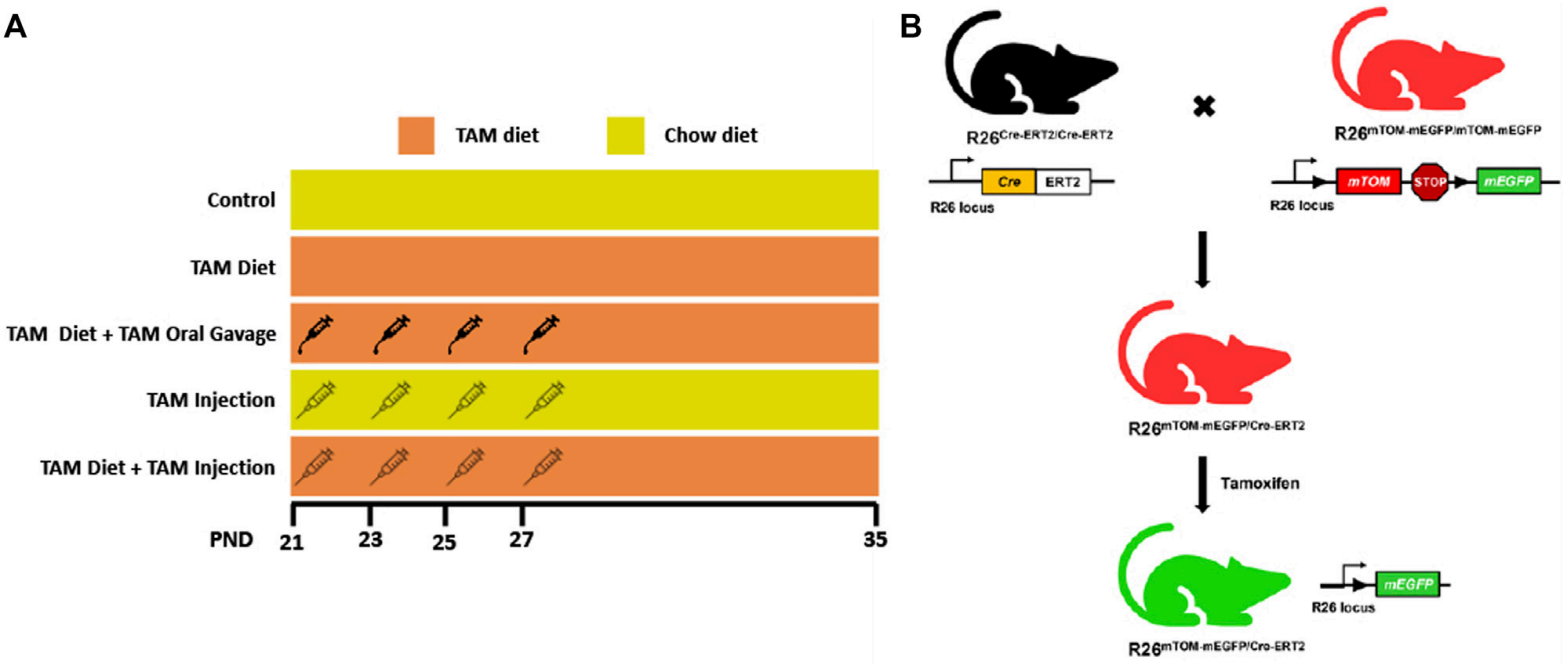
Schematic showing different tamoxifen (TAM) dosing regimens and the generation of TAM-inducible whole body reporter mice. **(A)** The schematic shows different TAM regimens used in the study. The control and TAM-treated mice received either 4 injections/gavages of corn oil or 4 injections/gavages of TAM in corn oil (1.35 mg/injection or gavage), respectively on alternate days starting postnatal day (PND) 21. The respective groups were maintained either on a TAM-diet (400 mg TAM-citrate/Kg food) or standard chow from PND 21 to 35. **(B)** Mice expressing LoxP-flanked *mTOM* stop cassette and a downstream *mEGFP* transgene under R26 locus were bred with mice expressing R26-regulated Cre recombinase gene fused with the gene encoding ligand-binding domain (LBD) of human estrogen receptor T2 (Cre-ERT2) to generate F1 progeny expressing single copies of Cre-ERT2 and mTOM/mEGFP transgene (R26^Cre−ERT2/mTOM-mEGFP^). TAM-mediated Cre induction leads to global mEGFP expression in these mice.

To determine the relative efficiency of Cre recombination through different routes and regimens of tamoxifen (TAM) administration (Figure 1A), we generated a reporter mice line expressing Cre-ERT2 and fluorescent reporters, mTOM and/or mEGFP in all cells and tissues (Figure 1B). A male mouse expressing Cre-ERT2 under R26 locus was crossed with a female reporter mouse carrying mTOM/mEGFP cassette (mT/mG mice) under R26 locus. The F1 mice carrying a single allele for each Cre-ERT2 and mT/mG transgene cassette (R26^Cre−ERT2/mTOM-mEGFP^) were used for the experiments.

### 3.2 Effects of tamoxifen treatment on body weight

**FIGURE 2:**
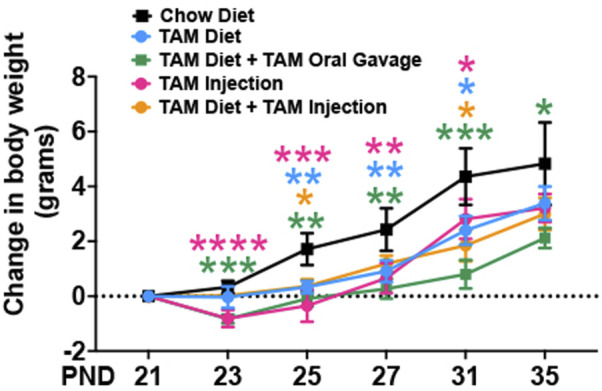
Time trend of change in body weight of mice subjected to different tamoxifen regimens and those that were fed a standard chow diet. Statistical analysis was performed by two-way ANOVA followed by Tukey’s multiple-comparison post hoc test. Data are represented as mean ± SEM. *p < 0.05, **p < 0.01, ***p < 0.001, ****p < 0.0001. *N* = 6–10 mice per group.

Tamoxifen treatment has been reported to be stressful to mice and often results in body weight loss (Kiermayer et al., 2007; Chiang et al., 2010; Miro-Murillo et al., 2011). To characterize the effects of tamoxifen administration on body weight, we assessed the body weights of mice on different TAM regimens throughout the treatment period, including in age-matched mice that were fed with a standard chow diet. The control group that was kept on a standard chow showed a progressive increase in body weight throughout the observation period. The TAM treatment resulted in a transient weight loss in mice of all groups followed by a progressive recovery (Figure 2). Of all the groups, mice that were kept on a combined TAM-diet plus TAM-oral gavage showed the slowest rate of weight gain when compared to mice that were kept on a standard chow. This change was statistically significant at all time points. We also observed a mortality rate of ∼10% (1/10) in this group.

### 3.3 Tamoxifen induces efficient Cre-mediated recombination in diverse tissues of mice

#### 3.3.1 Liver

**FIGURE 3:**
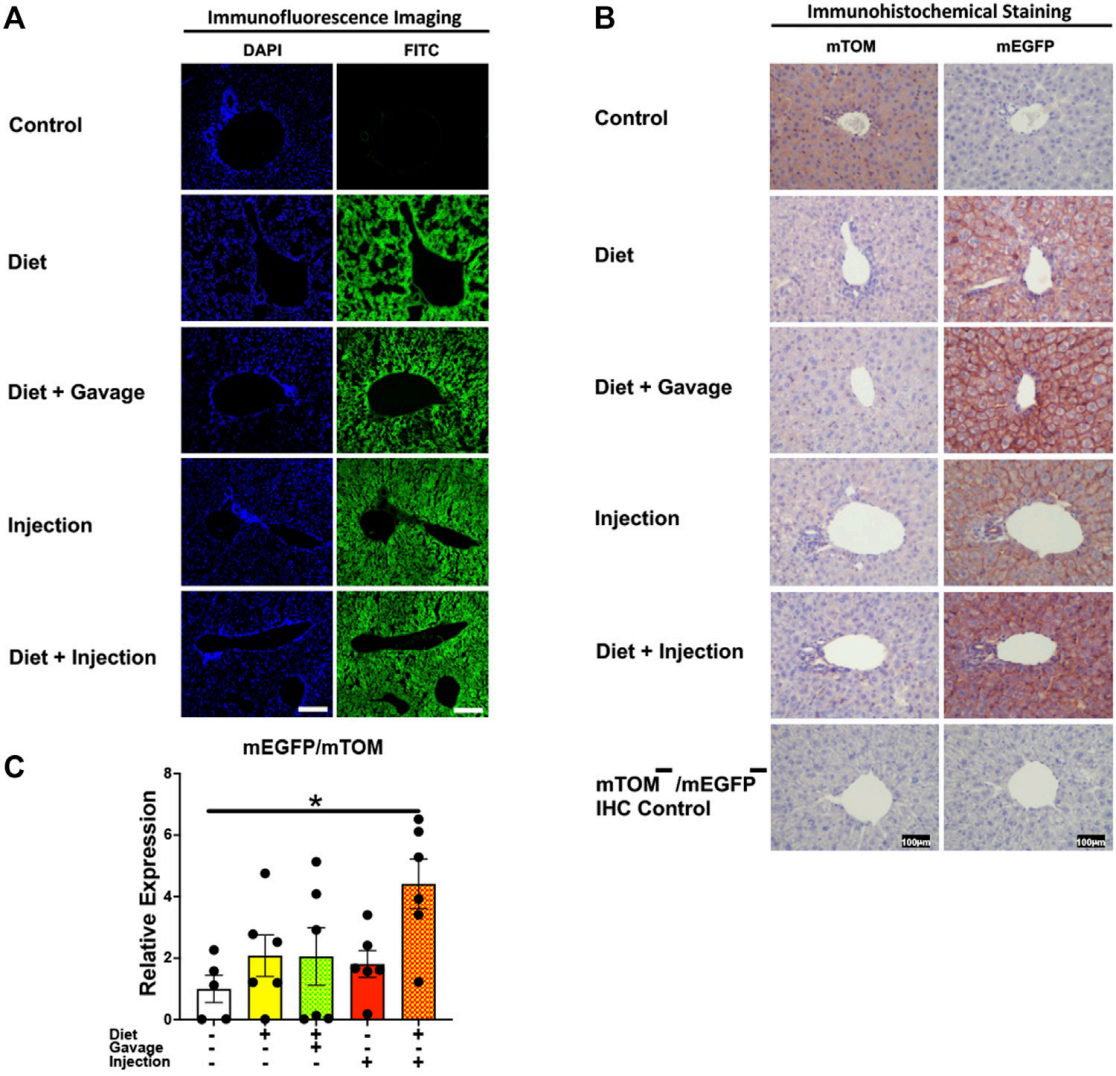
Inducible Cre-mediated expression of membrane-localized enhanced GFP (mEGFP) in the liver. **(A)** Representative fluorescence photomicrographs of fixed liver sections of R26^Cre−ERT2/mTOM-mEGFP^ mice subjected to different TAM-dosing regimens: TAM-diet (*N* = 6), TAM-diet plus TAM-oral gavage (*N* = 6), TAM-injections (*N* = 8), and TAM-diet plus TAM-injections (*N* = 8) showing complete mEGFP labeling in the hepatocytes. Green fluorescence was not evident in the liver of control mice fed a regular chow diet (*N* = 4). Scale bars: 170 µm. **(B)** Representative immunohistochemical photomicrographs of liver sections of R26^Cre−ERT2/mTOM-mEGFP^ mice subjected to different TAM-dosing regimens: TAM-diet (*N* = 5), TAM-diet plus TAM-oral gavage (*N* = 5), TAM-injections (*N* = 5), and TAM-diet plus TAM-injections (*N* = 5) showing complete mEGFP labeling in the hepatocytes. Widespread mTOM labeling was detected in the liver of regular chow diet-fed control mice (*N* = 5). The mTOM^−^/mEGFP^−^ mice (*N* = 4) lacking reporter transgene were included as IHC controls to ascertain the absence of mTOM or mEGFP staining. **(C)** Quantitative real-time RT-PCR showing the relative expression of m*EGFP*/m*TOM* in liver homogenate from the four TAM treatments and control groups. Statistical analysis was performed by one-ANOVA followed by Tukey’s multiple-comparison post hoc test. Data are represented as mean ± SEM. *p < 0.05. *N* = 5–6.

Tamoxifen undergoes biotransformation to a more potent metabolite, 4-hydroxytamoxifen, in the liver that mediates the translocation of Cre-ERT2 from the cytoplasm to the nucleus (Indra et al., 1999). As expected, the control regular chow-fed mice did not show any signs of mEGFP expression (Figures 3A,B). Further, the TAM-treated mice showed widespread mEGFP expression with varied fluorescence (Figure 3A) or immunochemical staining intensity (Figure 3B) in the liver regardless of the treatment regimen. Interestingly, however, the recombination in the portal region was variable among the treatment groups. The incidence of complete recombination in the portal region was highest in mice that were placed on a combined TAM-diet plus TAM-injection regimen (5/8) where the complete recombination was evident by mEGFP expression in the bile duct epithelium and portal vein endothelium, in addition to hepatocytes. Similar findings were noticed in the liver of one mouse on a combined TAM-diet and TAM-oral gavage regimen (1/6). mEGFP expression was essentially completely absent in the bile duct epithelium and other cells in the portal area of the mice that were solely maintained either on the TAM-diet or administered with TAM-injections alone (Figure 3B). In general, Cre recombination was very efficient in the hepatocytes of TAM-treated mice, regardless of the regimen, dose, and the route of TAM administration. However, Cre recombination in the bile duct epithelium and other cells in the portal region, including possibly fibroblasts and endothelial cells appeared to be dependent on the dose and/or route of TAM administration. Finally, we determined the relative expression of *mEGFP* to *mTOM* transcripts to quantitate Cre-mediated recombination in the floxed alleles. As compared to the control group, all four TAM treatment groups had increased expression of *mEGFP* transcripts (Figure 3C). However, among the four treatment regimens, only the combined TAM-diet and TAM-injection group showed significantly increased *mEGFP* expression compared to the control group (Figure 3C).

#### 3.3.2 Lungs

**FIGURE 4:**
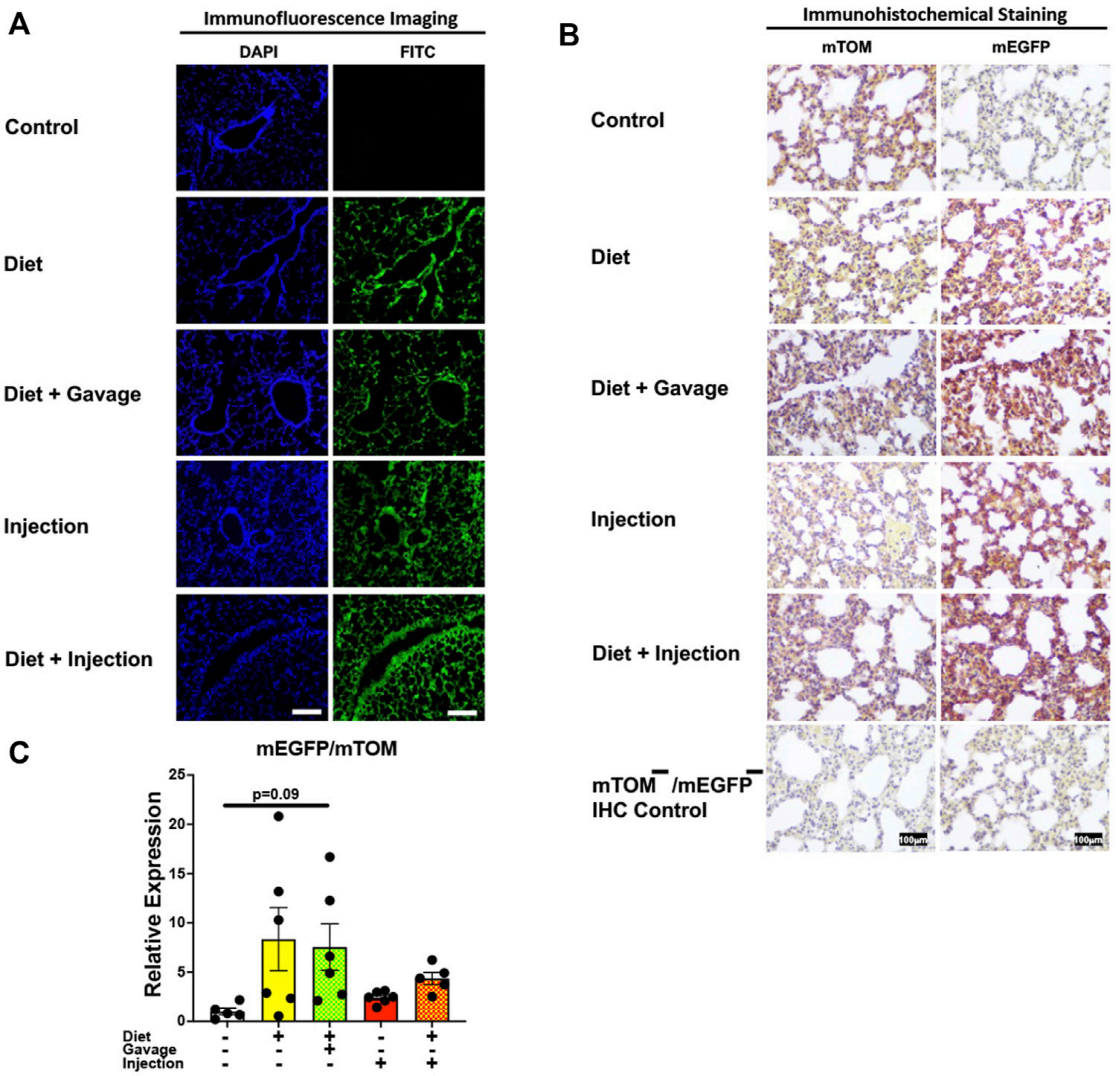
Inducible Cre-mediated expression of membrane-localized enhanced GFP (mEGFP) in the lungs. **(A)** Representative photomicrographs of fixed lungs sections of R26^Cre−ERT2/mTOM-mEGFP^ mice subjected to different TAM-dosing regimens: TAM-injections (*N* = 8), TAM-diet (*N* = 6), TAM-diet plus TAM-injections (*N* = 7), and TAM-diet plus TAM-oral gavage (*N* = 4) showing complete mEGFP labeling. Green fluorescence was not evident in the lungs of control mice fed a regular chow diet (*N* = 4). Scale bars: 170 µm. **(B)** Representative immunohistochemical photomicrographs of lungs sections of R26^Cre−ERT2/mTOM-mEGFP^ mice subjected to different TAM-dosing regimens: TAM-diet (*N* = 5), TAM-diet plus TAM-oral gavage (*N* = 5), TAM-injections (*N* = 5), and TAM-diet plus TAM-injections (*N* = 5) showing complete mEGFP labeling. Widespread mTOM labeling was detected in the lungs of regular chow diet-fed control mice (*N* = 5). The mTOM^−^/mEGFP^−^ mice (*N* = 4) lacking reporter transgene were included as IHC controls to ascertain the absence of mTOM or mEGFP staining. **(C)** Quantitative real-time RT-PCR showing the relative expression of m*EGFP*/m*TOM* in lungs homogenate from the four TAM treatments and control groups. Statistical analysis was performed by one-ANOVA followed by Tukey’s multiple-comparison post hoc test. *N* = 5–6.

Next, we analyzed Cre-ERT2-mediated recombination in the lungs of mice on different TAM regimens. TAM administration resulted in mEGFP expression in the airway and alveolar epithelial cells of all mice observed: combined TAM-diet plus TAM-injections (7/7), combined TAM-diet plus TAM-oral gavage (4/4), TAM-diet only (4/6), and TAM-injections only (6/8) (Figure 4A). The recombination appeared to be complete, as evident by robust expression of mEGFP, in all of the TAM regimens tested (Figure 4A). The control animals did not show any mEGFP expression in all the cells of the lungs. The immunohistochemical staining confirmed that the mEGFP expression is absent in the control mice and that the mEGFP expression is evident in all four TAM-treatment groups. However, the signal for mTOM persisted in all four TAM-treatment groups (Figure 4B) suggesting a slow turnover of mTOM protein that was translated before the recombination event took place. While the relative expression levels of *mEGFP* to *mTOM* transcripts were found to be higher in all TAM-treatment groups, the differences were not statistically significant as compared to the control group (Figure 4C).

#### 3.3.3 Heart

**FIGURE 5:**
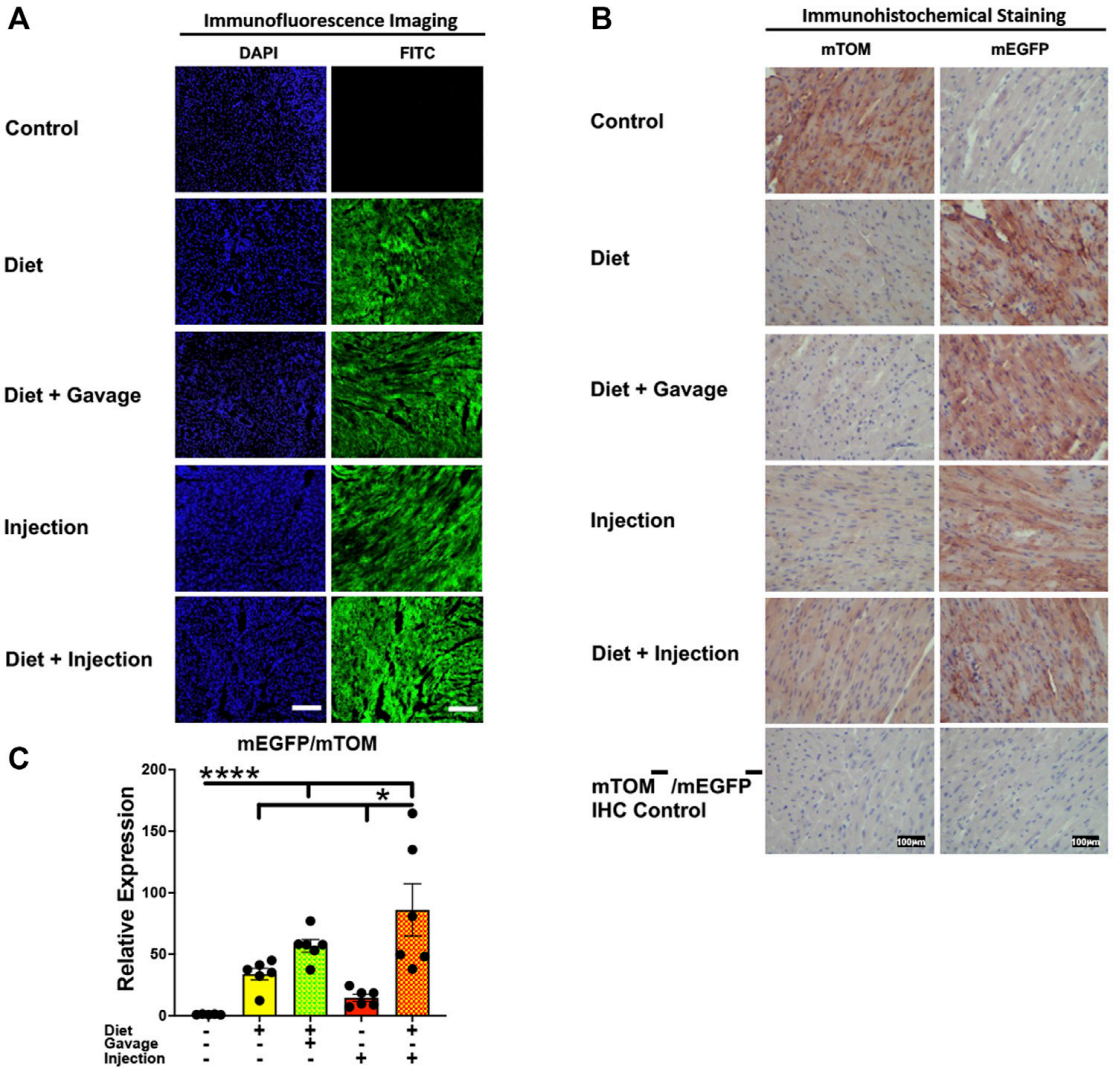
Inducible Cre-mediated expression of membrane-localized enhanced GFP (mEGFP) in the heart. **(A)** Representative photomicrographs of fixed heart sections of R26^Cre−ERT2/mTOM-mEGFP^ mice subjected to different TAM-dosing regimens: TAM-injections (*N* = 8), TAM-diet (*N* = 6), TAM-diet plus TAM-injections (*N* = 8), and TAM-diet plus TAM-oral gavage (*N* = 6) showing near-complete mEGFP labeling. Green fluorescence was not evident in the heart of control mice fed a regular chow diet (*N* = 4). Scale bars: 170 µm. **(B)** Representative immunohistochemical photomicrographs of heart sections of R26^Cre−ERT2/mTOM-mEGFP^ mice subjected to different TAM-dosing regimens: TAM-diet (*N* = 5), TAM-diet plus TAM-oral gavage (*N* = 5), TAM-injections (*N* = 5), and TAM-diet plus TAM-injections (*N* = 5) showing complete mEGFP labeling in the cardiac myofibers. Widespread mTOM labeling was detected in the heart of regular chow diet-fed control mice (*N* = 5). The mTOM^−^/mEGFP^−^ mice (*N* = 4) lacking reporter transgene were included as IHC controls to ascertain the absence of mTOM or mEGFP staining. **(C)** Quantitative real-time RT-PCR showing the relative expression of m*EGFP*/m*TOM* in heart homogenate from the four TAM treatments and control groups. Statistical analysis was performed by one-ANOVA followed by Tukey’s multiple-comparison post hoc test. Data are represented as mean ± SEM. *p < 0.05, ****p < 0.0001. *N* = 5–6.

Efficient Cre recombination was observed in the heart. Near complete recombination was observed in the cardiac myofibers of mice maintained on combined TAM-diet and TAM-injections (4/8) and those that were placed on combined TAM-diet and TAM-oral gavage (3/6) (Figures 5A,B). While mEGFP expression was observed in all the cardiac myofibers, weak signals for mTOM were also noticed (Figure 5B). The relative expression levels of *mEGFP* to *mTOM* transcripts were found to be higher in all TAM-treatment groups, however, the combination TAM-treatment groups, i.e., TAM-diet plus TAM-oral gavage and TAM-diet plus TAM-injections, showed a significant increase in *mEGFP* expression compared to the control group (Figure 5C).

#### 3.3.4 Intestine

**FIGURE 6:**
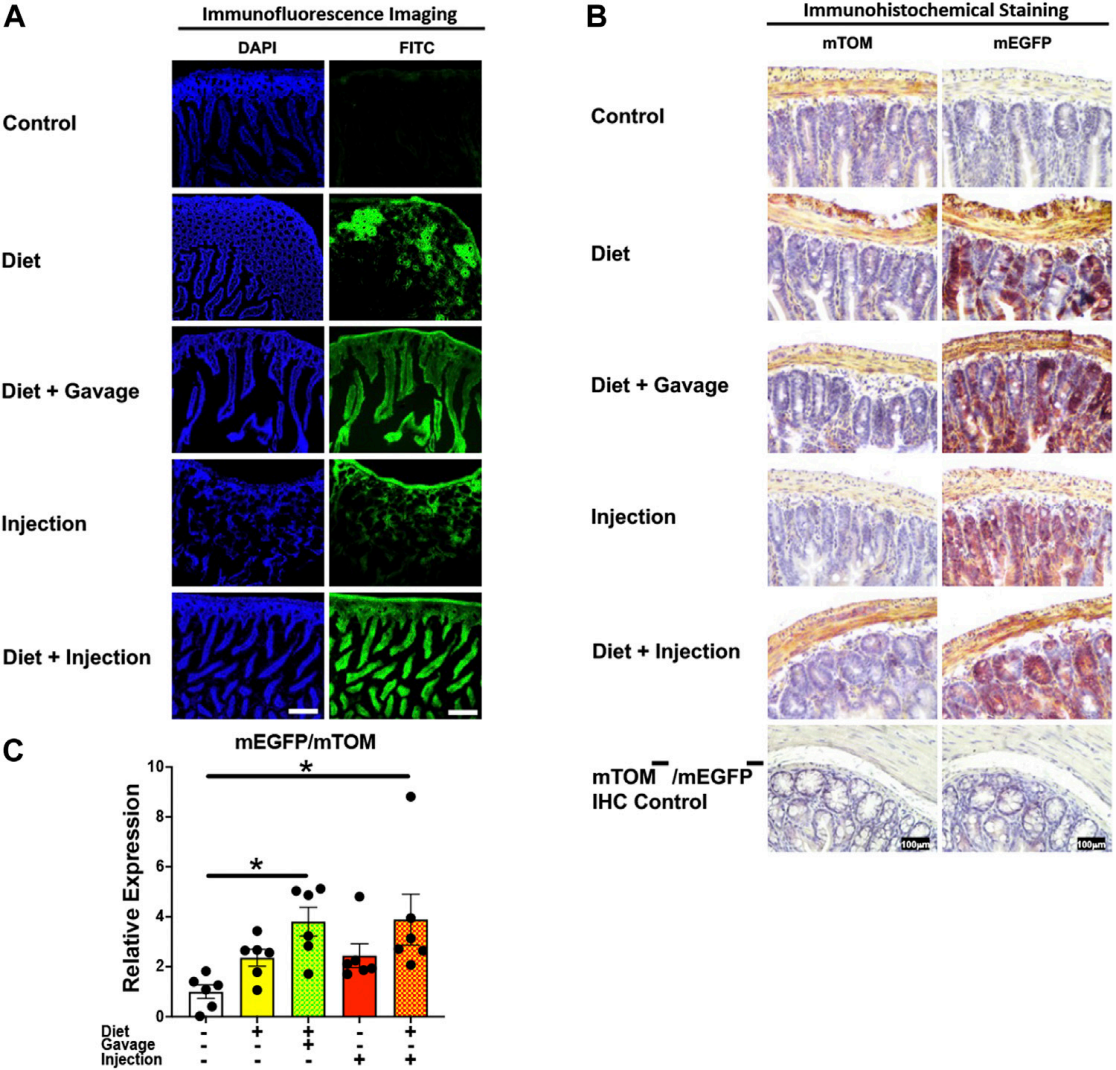
Inducible Cre-mediated expression of membrane-localized enhanced GFP (mEGFP) in the small intestine. **(A)** Representative photomicrographs of fixed small intestine sections of R26^Cre−ERT2/mTOM-mEGFP^ mice subjected to different TAM-dosing regimens: TAM-injections (*N* = 7), TAM-diet (*N* = 6), TAM-diet plus TAM-injections (*N* = 8), and TAM-diet plus TAM-oral gavage (*N* = 4) showing partial to complete mEGFP labeling. Green fluorescence was not evident in the small intestine of control mice fed a regular chow diet (*N* = 3). Scale bars: 170 µm. **(B)** Representative immunohistochemical photomicrographs of small intestine sections of R26^Cre−ERT2/mTOM-mEGFP^ mice subjected to different TAM-dosing regimens: TAM-diet (*N* = 5), TAM-diet plus TAM-oral gavage (*N* = 5), TAM-injections (*N* = 5), and TAM-diet plus TAM-injections (*N* = 5) showing partial to complete mEGFP labeling. mTOM labeling was detected in the small intestine of regular chow diet-fed control mice (*N* = 5). The mTOM−/mEGFP− mice (*N* = 4) lacking reporter transgene were included as IHC controls to ascertain the absence of mTOM or mEGFP staining. **(C)** Quantitative real-time RT-PCR showing the relative expression of m*EGFP*/m*TOM* in small intestine homogenate from the four TAM treatments and control groups. Statistical analysis was performed by one-ANOVA followed by Tukey’s multiple-comparison post hoc test. Data are represented as mean ± SEM. *p < 0.05. *N* = 5–6.

The mEGFP expression in the small intestine was variable among different TAM groups (Figure 6). The mice that were placed on TAM-diet either in combination with TAM-injections (7/8) or TAM-oral gavage (4/6) showed nearly complete recombination with mEGFP expression in the intestinal epithelium. However, the recombination was mostly incomplete in other layers of the intestinal wall including the submucosa, muscularis, and serosa. Interestingly, mEGFP expression was incomplete in the intestinal epithelium of mice maintained on the TAM-diet alone (3/6). Likewise, the recombination was incomplete in the submucosa, muscularis, and serosa in this group. Similarly, mice administered with TAM-injections only (7/7) showed incomplete Cre recombination in the intestinal epithelium and rare recombination in other layers of the intestinal wall. Interestingly, the lamina propria of the intestinal mucosa showed partial Cre activity in all TAM-treatment groups. The combination TAM-treatment groups, i.e., the TAM-diet plus TAM-oral gavage and TAM-diet plus TAM-injections, showed significantly increased *mEGFP* expression compared to the control groups (Figure 6C). These findings show that the combined TAM regimen is more effective at inducing Cre recombination in the small intestine.

#### 3.3.5 Lymphoid organs

**FIGURE 7:**
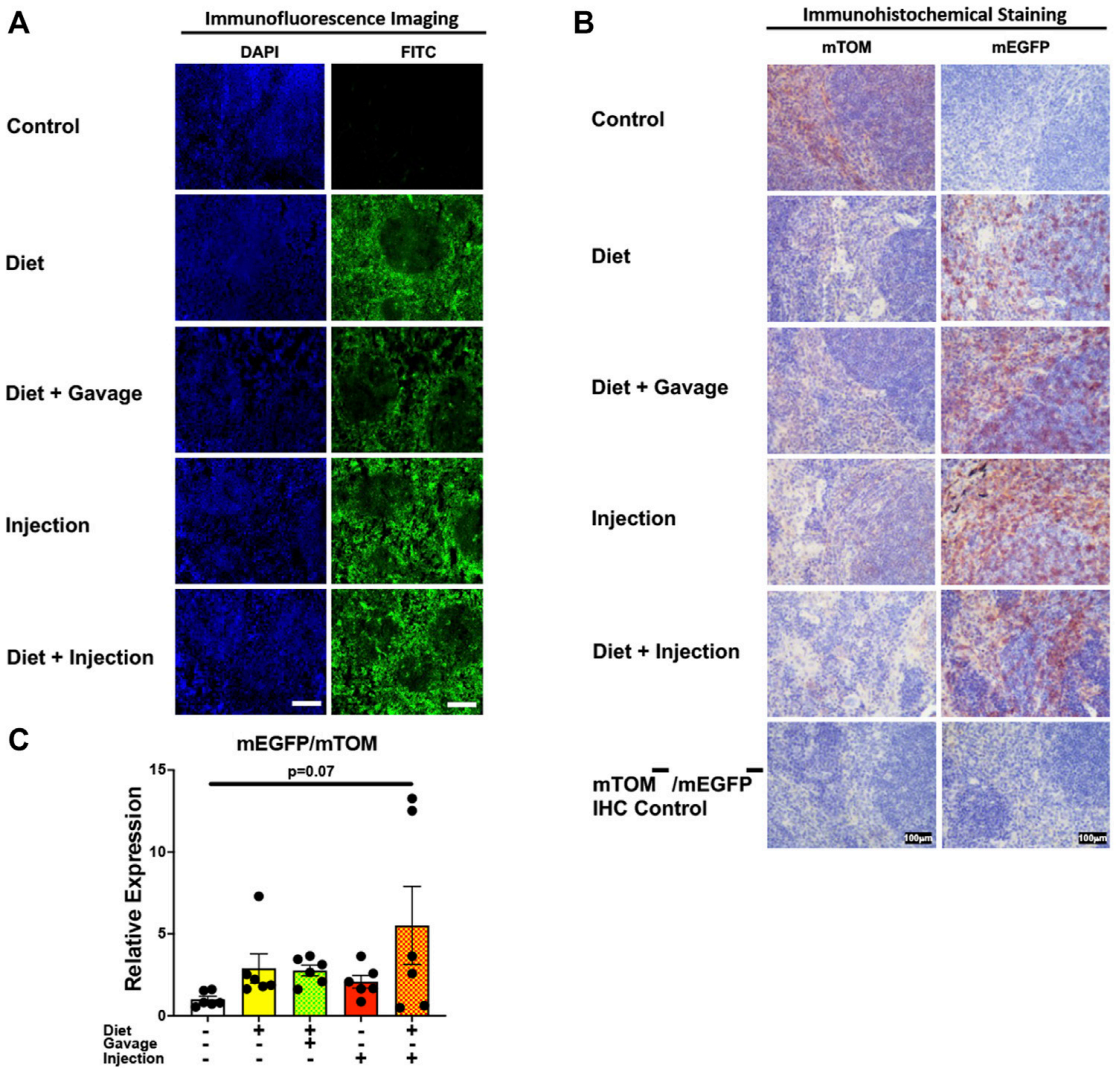
Inducible Cre-mediated expression of membrane-localized enhanced GFP (mEGFP) in the spleen. **(A)** Representative photomicrographs of fixed spleen sections of R26^Cre−ERT2/mTOM-mEGFP^ mice subjected to different TAM-dosing regimens: TAM-injections (*N* = 6), TAM-diet (*N* = 6), TAM-diet plus TAM-injections (*N* = 8), and TAM-diet plus TAM-oral gavage (*N* = 4) showing complete mEGFP labeling. Green fluorescence was not evident in the spleen of control mice fed a regular chow diet (*N* = 4). Scale bars: 170 µm. **(B)** Representative immunohistochemical photomicrographs of spleen sections of R26^Cre−ERT2/mTOM-mEGFP^ mice subjected to different TAM-dosing regimens: TAM-diet (*N* = 5), TAM-diet plus TAM-oral gavage (*N* = 5), TAM-injections (*N* = 5), and TAM-diet plus TAM-injections (*N* = 5) showing complete mEGFP labeling. Widespread mTOM labeling was detected in the spleen of regular chow diet-fed control mice (*N* = 5). The mTOM^−^/mEGFP^−^ mice (*N* = 4) lacking reporter transgene were included as IHC controls to ascertain the absence of mTOM or mEGFP staining. **(C)** Quantitative real-time RT-PCR showing the relative expression of m*EGFP*/m*TOM* in spleen homogenate from the four TAM treatments and control groups. Statistical analysis was performed by one-ANOVA followed by Tukey’s multiple-comparison post hoc test. *N* = 5–6.

**FIGURE 8:**
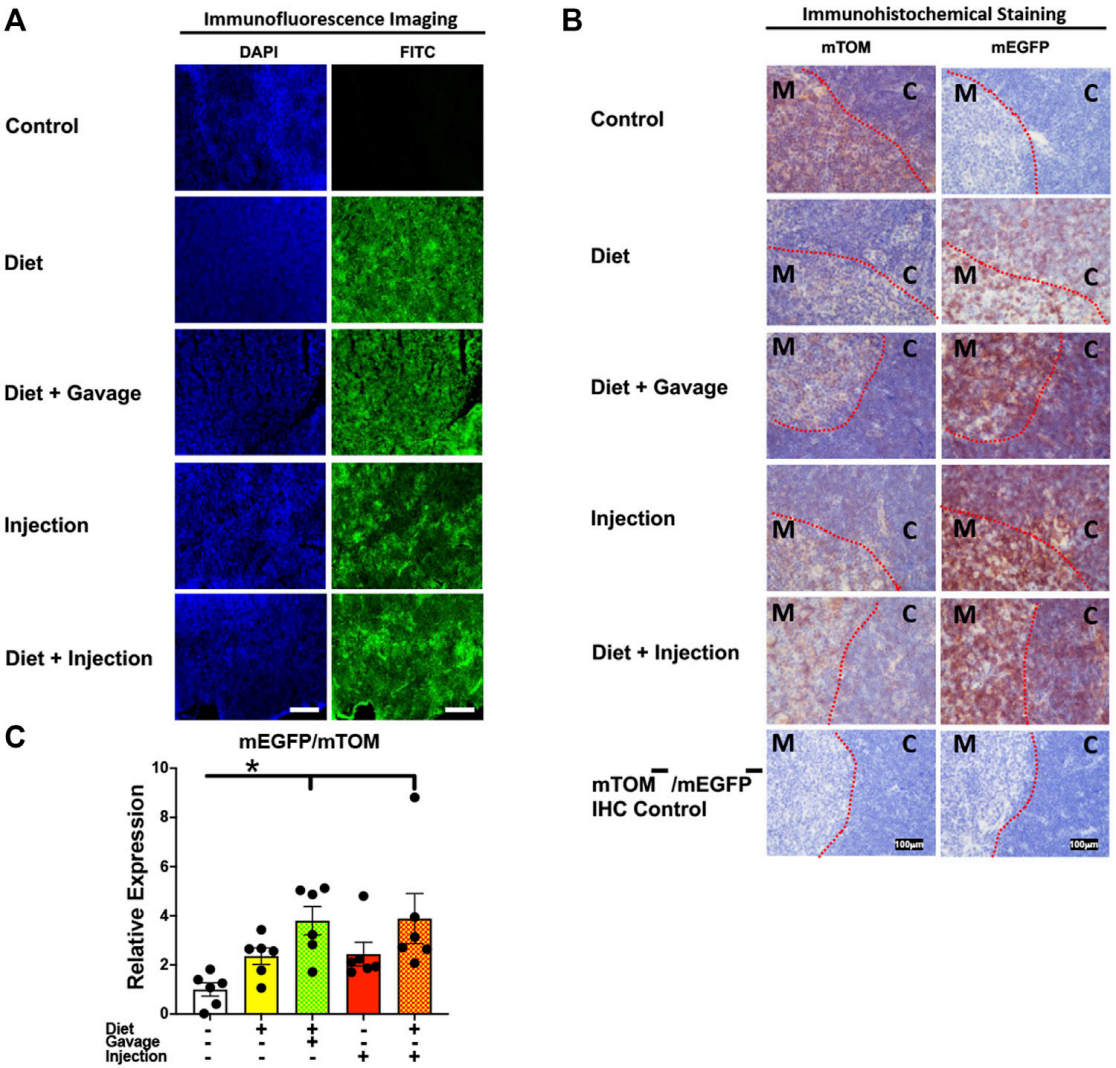
Inducible Cre-mediated expression of membrane-localized enhanced GFP (mEGFP) in the thymus. **(A)** Representative photomicrographs of fixed thymus sections of R26^Cre−ERT2/mTOM-mEGFP^ mice subjected to different TAM-dosing regimens: TAM-injections (*N* = 6), TAM-diet (*N* = 6), TAM-diet plus TAM-injections (*N* = 8), and TAM-diet plus TAM-oral gavage (*N* = 4) showing complete mEGFP labeling. Green fluorescence was not evident in the thymus of control mice fed a regular chow diet (*N* = 4). Scale bars: 170 µm. **(B)** Representative immunohistochemical photomicrographs of thymus sections of R26^Cre−ERT2/mTOM-mEGFP^ mice subjected to different TAM-dosing regimens: TAM-diet (*N* = 5), TAM-diet plus TAM-oral gavage (*N* = 5), TAM-injections (*N* = 5), and TAM-diet plus TAM-injections (*N* = 5) showing complete mEGFP labeling in the medulla (M) and cortex (C). Widespread mTOM labeling was detected in the thymus of regular chow diet-fed control mice (*N* = 5). The mTOM^−^/mEGFP^−^ mice (*N* = 4) lacking reporter transgene were included as IHC controls to ascertain the absence of mTOM or mEGFP staining. The dotted red lines indicate boundary between medulla and cortex. **(C)** Quantitative real-time RT-PCR showing the relative expression of m*EGFP*/m*TOM* in thymus homogenate from the four TAM treatments and control groups. Statistical analysis was performed by one-ANOVA followed by Tukey’s multiple-comparison post hoc test. Data are represented as mean ± SEM. *p < 0.05. *N* = 5–6.

To assess the inducible Cre activation in lymphoid organs, we examined mEGFP expression in the spleen and the thymus. Spleen showed complete recombination with strong green fluorescence (mEGFP) in both the white and the red pulp regions in all TAM-treatment regimens: combined TAM-diet plus TAM-injections (8/8), combined TAM-diet plus TAM-oral gavage (4/4), TAM-diet only (6/6) and TAM-injections only (6/6) (Figures 7A,B). Likewise, the thymus showed complete Cre recombination in both the cortex and the medulla in all TAM-treatment regimens: combined TAM-diet plus TAM-injections (4/4), combined TAM-diet plus TAM-oral gavage (6/6), TAM-diet only (3/3) and TAM-injections only (7/7) (Figures 8A,B). The relative expression levels of *mEGFP* to *mTOM* transcripts were found higher in all TAM-treatment groups in the spleen (Figure 7C) and thymus (Figure 8C). However, significant differences in *mEGFP* expression were only detected in the thymus in combined TAM-treatment groups, i.e., TAM-diet plus TAM-oral gavage and TAM-diet plus TAM-injections, which showed a significant increase in *mEGFP* expression compared to the control group (Figure 8C). Overall, these data suggest that Cre induction can be efficiently achieved by TAM administration through any of these TAM regimens, doses, and routes in the lymphoid tissues.

#### 3.3.6 Kidney

**FIGURE 9:**
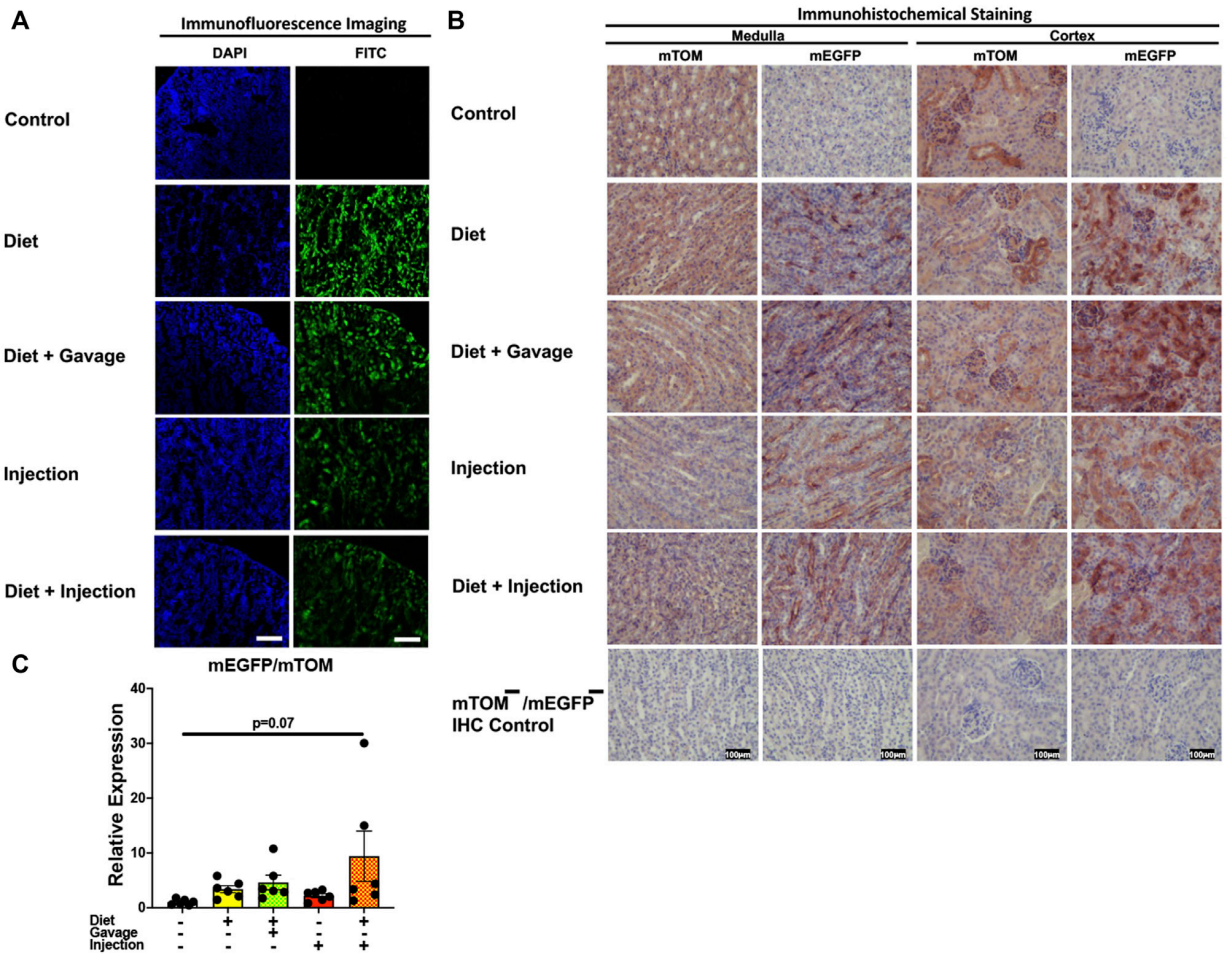
Inducible Cre-mediated expression of membrane-localized enhanced GFP (mEGFP) in the kidney: **(A)** Representative photomicrographs of fixed kidney sections of R26^Cre−ERT2/mTOM-mEGFP^ mice subjected to different TAM-dosing regimens: TAM-injections (*N* = 8), TAM-diet (*N* = 6), TAM-diet plus TAM-injections (*N* = 8), and TAM-diet plus TAM-oral gavage (*N* = 6) showing partial mEGFP labeling. Green fluorescence was not evident in the kidney of control mice fed a regular chow diet (*N* = 4). Scale bars: 170 µm. **(B)** Representative immunohistochemical photomicrographs of kidney medulla and cortex of R26^Cre−ERT2/mTOM-mEGFP^ mice subjected to different TAM-dosing regimens: TAM-diet (*N* = 5), TAM-diet plus TAM-oral gavage (*N* = 5), TAM-injections (*N* = 5), and TAM-diet plus TAM-injections (*N* = 5) showing complete mEGFP labeling. Widespread mTOM labeling was detected in the kidney of regular chow diet-fed control mice (*N* = 5). The mTOM^−^/mEGFP^−^ mice (*N* = 4) lacking reporter transgene were included as IHC controls to ascertain the absence of mTOM or mEGFP staining. **(C)** Quantitative real-time RT-PCR showing the relative expression of m*EGFP*/m*TOM* in kidney homogenate from the four TAM treatments and control groups. Statistical analysis was performed by one-ANOVA followed by Tukey’s multiple-comparison post hoc test. *N* = 5–6.

Interestingly, the kidney showed partial Cre recombination following TAM treatment. Regardless of the regimen and the route of TAM treatment, an incomplete Cre recombination was observed, particularly in the renal glomeruli but also in the renal tubules of all mice observed (Figures 9A,B). Additionally, the signal for mTOM persisted in the medulla and cortex of all four TAM-treatment groups (Figure 9B) suggesting a slow turnover of mTOM protein that was expressed prior to the recombination. The relative expression levels of *mEGFP* to *mTOM* transcripts were insignificantly higher in all TAM-treatment groups compared to the control group (Figure 9C).

#### 3.3.7 Skin

**FIGURE 10:**
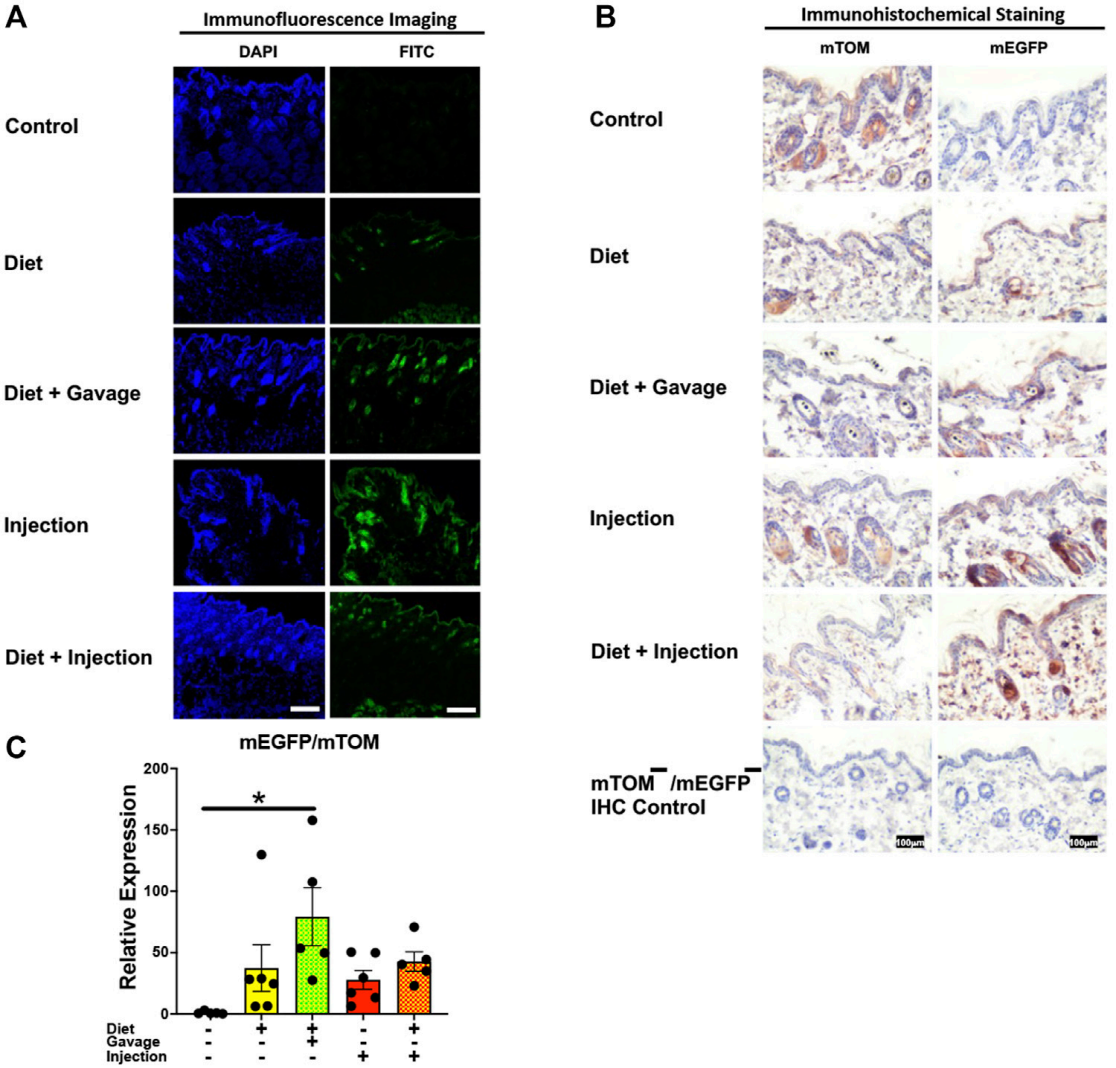
Inducible Cre-mediated expression of membrane-localized enhanced GFP (mEGFP) in the skin. **(A)** Representative photomicrographs of fixed skin sections of R26^Cre−ERT2/mTOM-mEGFP^ mice subjected to different TAM-dosing regimens: TAM-injections (*N* = 4), TAM-diet (*N* = 6), TAM-diet plus TAM-injections (*N* = 5), and TAM-diet plus TAM-oral gavage (*N* = 4) showing variable mEGFP labeling. Green fluorescence was not evident in the skin of control mice fed a regular chow diet (*N* = 3). Scale bars: 170 µm. **(B)** Representative immunohistochemical photomicrographs of skin sections of R26^Cre−ERT2/mTOM-mEGFP^ mice subjected to different TAM-dosing regimens: TAM-diet (*N* = 5), TAM-diet plus TAM-oral gavage (*N* = 5), TAM-injections (*N* = 5), and TAM-diet plus TAM-injections (*N* = 5) showing variable mEGFP labeling in the epidermis and hair follicles. Widespread mTOM labeling was detected in the skin of regular chow diet-fed control mice (*N* = 5). The mTOM^−^/mEGFP^−^ mice (*N* = 4) lacking reporter transgene were included as IHC controls to ascertain the absence of mTOM or mEGFP staining. **(C)** Quantitative real-time RT-PCR showing the relative expression of m*EGFP*/m*TOM* in skin homogenate from the four TAM treatments and control groups. Statistical analysis was performed by one-ANOVA followed by Tukey’s multiple-comparison post hoc test. Data are represented as mean ± SEM. *p < 0.05. *N* = 5–6.

Variable recombination was observed in the epidermis and hair follicles of the skin in TAM-treated mice (Figures 10A,B). Of all four TAM-treated groups, the combined TAM-diet plus TAM-oral gavage had minimal mTOM immunostaining suggesting a relatively higher degree of recombination (Figure 10B). As compared to the control diet group, while the *mEGFP* transcript expression was higher in all TAM-treated groups (Figure 10C), only the TAM-diet plus TAM-oral gavage group showed significantly higher *mEGFP* expression (Figure 10C).

#### 3.3.8 Brain

**FIGURE 11:**
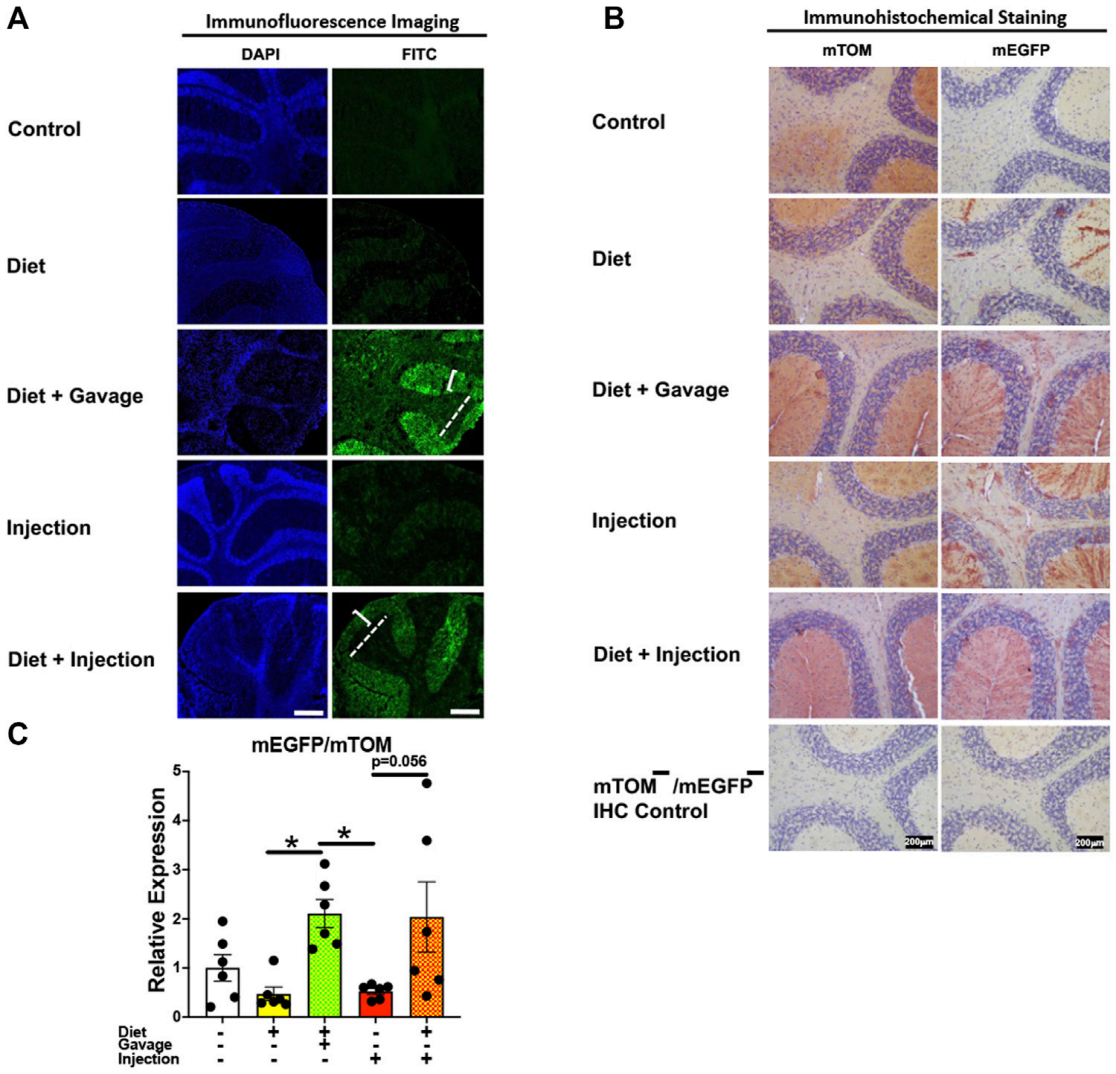
Inducible Cre-mediated expression of membrane-localized enhanced GFP (mEGFP) in the brain (cerebellum). **(A)** Representative photomicrographs of fixed brain sections of R26^Cre−ERT2/mTOM-mEGFP^ mice subjected to different TAM-dosing regimens: TAM-injections (*N* = 6), TAM-diet (*N* = 6), TAM-diet plus TAM-injections (*N* = 5), and TAM-diet plus TAM-oral gavage (*N* = 6) showing variable mEGFP labeling in the cerebellum. Green fluorescence was not evident in the brain of control mice fed a regular chow diet (*N* = 3). The bracket and dashed lines depict the molecular and granular layers of the cerebellum, respectively. Scale bars: 430 µm. **(B)** Representative immunohistochemical photomicrographs of brain sections of R26^Cre−ERT2/mTOM-mEGFP^ mice subjected to different TAM-dosing regimens: TAM-diet (*N* = 5), TAM-diet plus TAM-oral gavage (*N* = 5), TAM-injections (*N* = 5), and TAM-diet plus TAM-injections (*N* = 5) showing variable mEGFP labeling in the cerebellum. Widespread mTOM labeling was detected in the brain of regular chow diet-fed control mice (*N* = 5). The mTOM^−^/mEGFP^−^ mice (*N* = 4) lacking reporter transgene were included as IHC controls to ascertain the absence of mTOM or mEGFP staining. **(C)** Quantitative real-time RT-PCR showing the relative expression of m*EGFP*/m*TOM* in brain homogenate from the four TAM treatments and control groups. Statistical analysis was performed by one-ANOVA followed by Tukey’s multiple-comparison post hoc test. Data are represented as mean ± SEM. *p < 0.05. *N* = 6.

Of the four TAM regimens tested, the mEGFP expression was higher in the cerebellum of mice maintained on combined TAM-diet plus TAM-injections (4/5) and combined TAM-diet plus TAM-oral gavage (6/6) (Figures 11A,B). Sporadic mEGFP expression was observed in the cerebellum of mice administered either with TAM-injections (5/6) only or TAM-diet alone (3/6) (Figures 11A,B). In general, the mEGFP expression was more prominent in the molecular layer than in the granular layer of the cerebellum. Consistent with the fluorescence and immunostaining for mEGFP signals (Figures 11A,B), the *mEGFP* transcript expression in the combined TAM-diet plus TAM-oral gavage group was significantly higher as compared to TAM-diet alone and TAM-injection alone group (Figure 11C).

## 4 Discussion

The inducible Cre-Lox recombination system has revolutionized our approach to studying cell- and tissue-specific functions of a particular gene at any developmental stage (Feil et al., 2009). Cre-ERT2 utilizes ERT2 ligand to induce Cre activity and thus offers temporal control over gene recombination. The binding of the 4-hydroxytamoxifen (4-OHT) ligand, an active metabolite of TAM, to the ligand-binding domain of Cre-ERT2 fusion protein allows the translocation of Cre into the nucleus. TAM is commonly administered through intraperitoneal and oral routes for Cre-ERT2 activation (Schuler et al., 2004; Kiermayer et al., 2007; Donocoff et al., 2020; Smith et al., 2022). However, the relative efficiency of gene recombination in various tissues using these routes of TAM administration are poorly characterized. Besides, the optimal TAM dose and the time course required to achieve effective gene deletion in various tissues are not known. Therefore, in this study, we aimed to standardize TAM-dosing regimens that would result in efficient Cre-mediated recombination within floxed genes in major body tissues.

Double fluorescent Cre reporter mice harboring mTOM/mEGFP transgene are widely used to determine the efficacy of Cre-mediated recombination within floxed genes and lineage tracing studies (Chandran et al., 2021; Forte et al., 2022; Klein et al., 2022). Thus, we utilized mTOM/mEGFP transgene to assess Cre recombination using green fluorescence and immunohistochemical localization of mEGFP, as end-points for protein expression, and the ratio of *mEGFP* to *mTOM* transgene expression, as an end-point for gene expression analysis. As reported previously, all examined tissues exhibited a strong red fluorescence (mTOM) before TAM administration (Muzumdar et al., 2007). TAM resulted in the induction of green fluorescence in all the tissues examined. The degree of Cre recombination in various tissues, however, was variable among different regimens of TAM administration. This is in line with previous studies that have shown that TAM-induced Cre recombination varies among different tissues and is dependent on the TAM dose (Guo et al., 2002; Hayashi and McMahon, 2002; Hameyer et al., 2007; Ichise et al., 2016). In most tissues, including the liver, heart, small intestine, thymus, and skin, TAM-diet in combination with either TAM-injections or TAM-oral gavage resulted in higher mEGFP expression, indicating that increased TAM dose is relatively more effective in inducing recombination. On rare occasions, we found variable recombination within the same group. This variability was much more frequent in mice that were placed on TAM-diet alone. The possible explanation for this observation is an unregulated intake of TAM-diet and a consequent variation in the bioavailability of active TAM metabolite, 4-hydroxytamoxifen, at the cellular level (Smith et al., 2022).

The recombination efficiency is greatly influenced by factors including the TAM dose, the route of TAM delivery, and the duration of the treatment (Hayashi and McMahon, 2002). A previous study reported that efficient Cre recombination in the liver (∼80% of hepatocytes) could be achieved by intraperitoneal administration of 1 mg TAM/day for 5-consecutive days in adult mice (Schuler et al., 2004). In this study, Cre-ERT2 was expressed under serum albumin promoter and these authors analyzed recombination 5-days after the last TAM injection. However, the status of recombination in the biliary epithelium and the portal vein endothelium remained unknown in this study (Schuler et al., 2004). In our study, we found an efficient Cre recombination in the hepatocytes (∼100% of hepatocytes) in all the TAM regimens tested. However, we noticed that of all the regimens, the TAM-diet in combination with TAM-injections, was the most effective in inducing recombination in the liver, including in the biliary epithelium, in addition to the hepatocytes. TAM-injections are an effective and controlled method of TAM delivery, however, with TAM-injections alone, the biliary epithelial cells and/or the portal vein endothelial cells appeared to have partial recombination. When combined with the TAM-diet, TAM-injections were extremely effective in inducing recombination in all cells including biliary epithelial cells and/or endothelial cells. This indicates that both the TAM dose and the route of TAM delivery are critical in inducing effective recombination in this tissue.

The dose-dependent Cre activity is reported in a previous study that showed that 2.4 mg TAM was better than 1.2 mg TAM in inducing recombination in CD45^+^ cells (Donocoff et al., 2020). These authors also showed that the intraperitoneal (IP) route was better than the oral route of TAM delivery. Interestingly, some studies have shown that the oral route of TAM delivery is comparable to and sometimes better than the injection route (Forde et al., 2002; Andersson et al., 2010; Yoshinobu et al., 2021). However, these authors used a comparatively higher TAM concentration in the diet (0.5–2.5 mg TAM/g feed) than used in our study (0.4 mg TAM/g feed). Moreover, as compared to TAM-citrate present in the TAM-diet, these authors used the free base form of TAM, which is more efficient in inducing recombination (Andersson et al., 2010). This may be another reason for the suboptimal recombination observed in mice put on the TAM-diet alone in our study. Besides the dose and the route of TAM delivery, the duration of TAM treatment can have a considerable influence on the efficiency of recombination. We noticed sub-optimal recombination in the endothelium at the portal areas regardless of the route of TAM administration. A previous study compared the duration of TAM administration to achieve endothelial cell-specific Cre recombination in Tie2^Cre−ERT2^; RAGE/EGFP (RA/EG) reporter mice (Forde et al., 2002). The authors reported that feeding mice with a tamoxifen mixed diet (2.5 mg/g feed) for 5 weeks, but not 3 weeks, resulted in satisfactory endothelial cell-specific recombination in organs including heart, lungs, liver, spleen, kidney, and pancreas. This shows the inherently slow reactivity of endothelial cells to TAM.

Cre recombination appeared to be dose-dependent in cardiac myocytes, i.e., more in combined TAM-treatment regimens and less in TAM-diet or TAM-injection alone groups. Interestingly, in the kidney, the recombination appeared to be unaffected by the TAM regimen, TAM dose, and the route of TAM administration. The renal tubular epithelial cells and the renal glomeruli both showed partial recombination in all TAM-treatment regimens. A previous study compared oral and intraperitoneal routes of TAM administration to attain renal epithelial cell-specific Cre recombination using KspCad^Cre−ERT2^; lacZ/EGFP (Z/EG) reporter mice (Lantinga-van Leeuwen et al., 2006). These authors administered either 5 mg TAM/day through oral gavage or 2 mg TAM/day through intraperitoneal injections for 5-consecutive days and analyzed the kidney sections 7 days post-last TAM-treatment. These TAM doses are much higher than what we used in the current study. However, despite using a higher TAM dose, these authors reported a mosaic expression of EGFP/green fluorescence in the renal tubular epithelial cells and the absence of EGFP expression in the glomeruli. Moreover, the recombination efficiency was found to be comparable between the two dosing routes. In contrast, we observed partial recombination in both the glomeruli and the renal tubular epithelial cells, although the recombination was somewhat lower in the glomeruli than in the renal tubular epithelial cells. A possible explanation for these variations between the two studies is the usage of different promoters to express Cre recombinase, KspCad versus Rosa 26. The Cre-ERT2 expression is restricted to the epithelial cells of the proximal and distal convoluted tubules, loop of Henle, and the collecting ducts under KspCad promoter while the expression is ubiquitous under Rosa 26.

The recombination efficiency was highest in the epidermis and hair follicles of the TAM-diet plus TAM-oral gavage group only. Our findings in the skin were somewhat comparable to a previous study that reported an efficient Cre recombination in the epidermis of mice expressing Cre-ERT2 under the control of human keratin 14 promoter (Indra et al., 2000). Interestingly, these authors injected a considerably low TAM dose (0.1 mg TAM/day for 5-consecutive days) and showed successful recombination in the keratinocytes of the epidermis and outer root sheath of hair follicles as early as 5 days after the start of TAM injections. In contrast, Hameyer et al. (2007) reported partial recombination in the skin of Rosa26-Cre-ERT2 LacZ reporter mice using a comparatively higher TAM dose (1 mg TAM/day for 5-consecutive days). Although, in this study, the status of recombination in various anatomical structures of the skin was not discussed.

The cerebellum showed minimal and partial Cre recombination in diet-only and injection-only groups, respectively. More efficient recombination was observed in combined TAM-dosing regimens (diet plus injection; diet plus oral gavage) indicating that Cre activation in the brain requires a relatively higher TAM dose or sustained levels of TAM in the blood. Limited permeability through the blood-brain barrier might be a probable cause for reduced levels of TAM in the brain (Jahn et al., 2018). Smith et al., showed that a 5-day consecutive TAM-injection regimen (180 mg/kg body weight/day IP; ∼4–5mg/dose) induces more efficient recombination versus a 2-week TAM diet (500 mg TAM/kg diet) in the hippocampus of Nestin^Cre−ERT2^/R26-LoxP-STOP-LoxP-EYFP reporter mice (Smith et al., 2022) indicating route-/dose-dependent effect. This is because voluntary intake of TAM diet-alone exposes mice to less overall TAM dose compared to involuntary TAM-injections. A previous study highlighted the importance of sustained levels of TAM and its metabolites on Cre activity in the brain (Jahn et al., 2018). These authors compared the efficiency of 3- or 5-day consecutive injections and 3 alternate-day injections (100 mg TAM/Kg body weight) in inducing recombination in astroglia of GLAST^CreERT2/+^/P2Y1^fl/fl^/GluA1^fl/fl^ young adult mice. They demonstrated that the alternate day protocol was less efficient than the consecutive day protocol due to the rapid clearance of TAM and its metabolites from the brain and the serum within 48 h of injection (Jahn et al., 2018). This explains the relatively inefficient Cre recombination in the brain of mice solely maintained on alternate TAM-injections in our study.

Besides the bioavailability of 4-OHT, certain tissue or cell-specific factors can affect recombination efficiency, especially in the reporter-based system. These include variations in the synthesis, trafficking, and turnover rates of fluorescent proteins in various tissues. As previously reported (Muzumdar et al., 2007), we observed variable mEGFP fluorescence across different tissues, regardless of the TAM regimen. These authors claimed that the persistence of mTOM in recombined cells contributes to the double-fluorescent cell population (yellow) in a tissue. For instance, they observed complete disappearance of red fluorescence in hepatocytes, 9 days after the first TAM-injection. Along similar lines, we observed complete mEGFP labeling in the hepatocytes in the current study. However, it appeared that mTOM persisted for a comparatively longer duration (2 weeks after the first TAM administration) in the heart, lungs, and kidney in our study. This shows that the cellular dynamics of reporter proteins can vary in different tissues and thus caution should be exercised especially when the interpretation of recombination efficiency is solely based on the reporter system.

TAM is known to induce physiological stress in mice and often results in transient weight loss (Kiermayer et al., 2007; Miro-Murillo et al., 2011). This was also evident in our study. All the groups showed transient weight loss at the beginning of the TAM dosing regimen, after which they started gaining weight, albeit slowly as compared to the group that was fed standard chow. Of all the groups, TAM-diet plus TAM-oral gavage group showed the slowest recovery at all the time points examined. The probable reasons for these observations include TAM-induced liver toxicity, and/or loss of body fat reservoirs (Zhao et al., 2020). Additionally, however, the stress of TAM administration *via* oral gavage may have led to poor/slow recovery in this particular group. The TAM-diet group appeared to be the least affected group indicating that this method is comparably less stressful and can be preferred in certain situations, such as in metabolic studies.

Cre activity can greatly influence the recombination efficiency and targeted gene deletion. Cre-ERT2 recombinase is reportedly more potent than its predecessor, Cre-ERT in driving the recombination (Indra et al., 1999). Indra et al. conducted a dose-response study to compare the efficiencies of Cre-ERT and Cre-ERT2 recombinases in the keratinocytes of double heterozygous K5^Cre−ERT^/ACZL and K5^Cre−ERT2^/ACZL mice, respectively. They compared three different doses of 4-OHT (0.01, 0.1, 1.0 mg) daily for 5 days and waited for 11-days post-last injection for analysis. These authors reported a higher (∼10-fold) sensitivity of Cre-ERT2 to 4-OHT, compared to Cre-ERT, and showed a successful nuclear translocation of Cre-ERT2, but not Cre-ERT, with the administration of 0.01 mg 4-OHT for five-consecutive days. These studies indicate that the low sensitivity of Cre-ERT is less likely to be compensated by the direct administration of a more potent tamoxifen metabolite, 4-OHT. Similarly, a previous study showed a 30% deletion in the liver of mice ubiquitously expressing Cre-ERT under the control of cytomegalovirus promoter (Feil et al., 1996). These authors administered 1 mg 4-OHT daily for 5 consecutive days IP and waited for 2 days for analysis post-last injection. Likewise, Imai et al. (2000) reported recombination in only ∼40%–50% of the hepatocytes after TAM treatment in mice expressing Cre-ERT under α1-antitrypsin promoter. In the current study, TAM resulted in an efficient Cre-ERT2 induction in multiple organs and thus allowed us to standardize different TAM-dosing regimens.

The activity of a promoter can also significantly influence the expression of an inserted transgene. R26 is a well-characterized gene locus that is ubiquitously active. However, its activity has been reported to be low in the central nervous system (Hameyer et al., 2007). In a previous study that used LacZ expression as a readout (Hameyer et al., 2007), the authors reported a failure of Cre-ERT2 expression under the R26 locus in the brain. In line with this study, we also noticed poor mEGFP expression in the brain in all TAM-administered mice except those that were placed on a combined regimen of TAM-diet either with TAM-injections or TAM-oral gavage that showed comparatively better recombination in the cerebellum. Interestingly, the mEGFP signal was somewhat intense in the molecular layer of the cerebellum. Muzumdar et al. also reported a similar finding in the cerebellum of Rosa26^mT/mG^ mice (Muzumdar et al., 2007). They speculated the likely enrichment of membrane-restricted EGFP in the axonal processes because of a larger surface-to-volume ratio leading to a greater signal in axon abundant molecular layer of the cerebellum.

There are a few limitations of this study that could be addressed in future studies. First, we were not able to test the effectiveness of longer durations of TAM treatment/time-course of TAM administration. Second, we were not able to access the efficiency of the TAM-dosing regimens in adult and aged mice. Third, we did not measure the serum and organ-specific concentration and degradation kinetics of TAM and its metabolites, including 4-hydroxytamoxifen, endoxifen, and norendoxifen, and their relation to recombination efficiency. Lastly, we did not validate the reporter-based recombination efficiency with the deletion of a specific floxed gene.

In conclusion, the present study compared different regimens and routes of TAM administration in driving whole-body inducible gene deletion using double fluorescent reporter mice. TAM administration resulted in successful Cre recombination to a variable extent in diverse tissues with combined TAM-diet and TAM-injections or combined TAM-diet and TAM-oral gavage being the most effective methods to achieve Cre-Lox recombination. Finally, we recommend that in future studies, various combinations of TAM doses, routes of TAM delivery, and durations of TAM treatment should be tested to further characterize temporal Cre recombination in different tissues. Moreover, we believe that there is a need to test more efficient cell-specific Cre-ERT2 reporter mice lines to better optimize spatial Cre recombination.

## Data Availability

The original contributions presented in the study are included in the article/Supplementary Material, further inquiries can be directed to the corresponding author.
